# Iron deposition in the precuneus is correlated with mild cognitive impairment in patients with cerebral microbleeds: A quantitative susceptibility mapping study

**DOI:** 10.3389/fnins.2022.944709

**Published:** 2022-08-08

**Authors:** Jing Tu, Jin Yan, Juan Liu, Dandan Liu, Xiaomeng Wang, Fei Gao

**Affiliations:** ^1^Department of Neurology, The First Affiliated Hospital of Xi'an Medical University, Xi'an, China; ^2^Xi'an Medical University, Xi'an, China; ^3^Department of Radiology, The First Affiliated Hospital of Xi'an Medical University, Xi'an, China

**Keywords:** cerebral small vessel disease, cerebral microbleed, cognitive impairment, precuneus, quantitative susceptibility mapping

## Abstract

**Purpose:**

The purpose of this study was to define whether mild cognitive impairment (MCI) is associated with iron deposition in rich-club nodes distant from cerebral microbleeds (CMBs) in patients with cerebral small vessel disease (CSVD).

**Methods:**

A total of 64 participants underwent magnetic resonance imaging (MRI) scanning and were separated into three groups, namely, CMB(+), CMB(–), and healthy controls (HCs). We compared their characteristics and susceptibility values of rich-club nodes [e.g., superior frontal gyrus (SFG), precuneus, superior occipital gyrus (SOG), thalamus, and putamen]. We then divided the CMB(+) and CMB(–) groups into subgroups of patients with or without MCI. Then, we analyzed the relationship between iron deposition and MCI by comparing the susceptibility values of rich-club nodes. We assessed cognitive functions using the Montreal Cognitive Assessment (MoCA) and quantified iron content using quantitative susceptibility mapping (QSM).

**Results:**

In the putamen, the CMB(+) and CMB(–) groups had significantly different susceptibility values. Compared with the HCs, the CMB(+) and CMB(–) groups had significantly different susceptibility values for the SFG and SOG. In addition, we found significant differences in the putamen susceptibility values of the CMB(+)MCI(+) group and the two CMB(–) groups. The CMB(+)MCI(+) and CMB(+)MCI(–) groups had significantly different precuneus susceptibility values. The binary logistic regression analysis revealed that only higher susceptibility values of precuneus were associated with a cognitive decline in patients with CMBs, and it indicated statistical significance.

**Conclusion:**

Iron deposition in the precuneus is an independent risk factor for MCI in patients with CMBs. CMBs might influence iron content in remote rich-club nodes and be relevant to MCI.

## Introduction

Age-related diseases have gained substantial significance with the rise in life expectancy (Kontis et al., 2017). The prevalence of mild cognitive impairment (MCI) in people over the age of 65 years is 20.8%, and 42.0% of the cases are induced by cerebrovascular diseases and vascular risk factors (Jia et al., 2014). Cerebral small vessel disease (CSVD) is one of the significant vascular risk factors for cognitive impairment and dementia (METACOHORTS Consortium, 2016) and has caused growing concern in recent years. Cerebral microbleeds (CMBs), a typical imaging marker of CSVD (Wardlaw et al., 2013), have an incidence of 5–35% in individuals aged older than 45 years (Haller et al., 2018). The proportion of CMBs is 16–45% and up to 86% in patients with cognitive impairment and vascular dementia, respectively (Boyano et al., 2018). A high CMB count might increase the risk of cognitive decline, even dementia, and the location of CMBs might involve the different impaired cognitive domains (Akoudad et al., 2016; Ding et al., 2017; Li et al., 2021).

Regarding the mechanisms underlying cognitive dysfunction, investigators have gradually shifted their interests from abnormal protein to abnormal iron concentration (Apostolakis and Kypraiou, 2017). Indeed, iron deposition plays a crucial role in cognitive impairment and structural injury (Schröder et al., 2013; Yang et al., 2021). Due to technological developments in magnetic resonance imaging (MRI), iron deposition is no longer confined to postmortem studies or animal experiments. Magnetic susceptibility methods can indirectly quantify iron. For example, quantitative susceptibility mapping (QSM), a new quantitative magnetic susceptibility technology, can quantify iron, calcification, and venous oxygen saturation *in vivo* (Haacke et al., 2015). In recent years, studies based on QSM have been burgeoning because this technique is not susceptible to the non-local field effects, contrary to other methods, such as phase values and R2^*^ (Persson et al., 2015).

Recently, Li et al. discovered that a higher iron burden of CMB focal was related to worse cognitive function. Meanwhile, they found iron deposition in certain deep gray matter areas of patients with CMB, which was in line with another study (Liu et al., 2016; Li et al., 2022). However, the reason why local CMBs impact higher cortical function has remained unknown. Brain structure networks analysis might shed new light on this mechanism. Researchers found that the effect of CMBs was not only local but also global because CMBs could alter white matter integrity (Akoudad et al., 2013; Liu et al., 2020). Tuladhar et al. (2017) proposed the areas such as the superior frontal gyrus (SFG), precuneus, superior occipital gyrus (SOG), thalamus, and putamen as rich-club nodes of CSVD. These structures are rich and densely connected (van den Heuvel and Sporns, 2011). Thus, rich-club nodes are considered the centers of the brain network, and they are responsible for information integration (van den Heuvel et al., 2012). Lesions located on rich-club nodes reduce their connectivity and impair cognitive performance (Fornito et al., 2015). Thus, we hypothesized that iron deposition in rich-club nodes probably mediated cognitive impairment in patients with CMB. In this study, we used QSM to (1) compare susceptibility values in rich-club nodes of patients with or without CMBs and (2) understand the relationship between MCI and iron deposition in rich-club nodes in patients with CMBs.

## Materials and methods

### Participants

**Figure 1:**
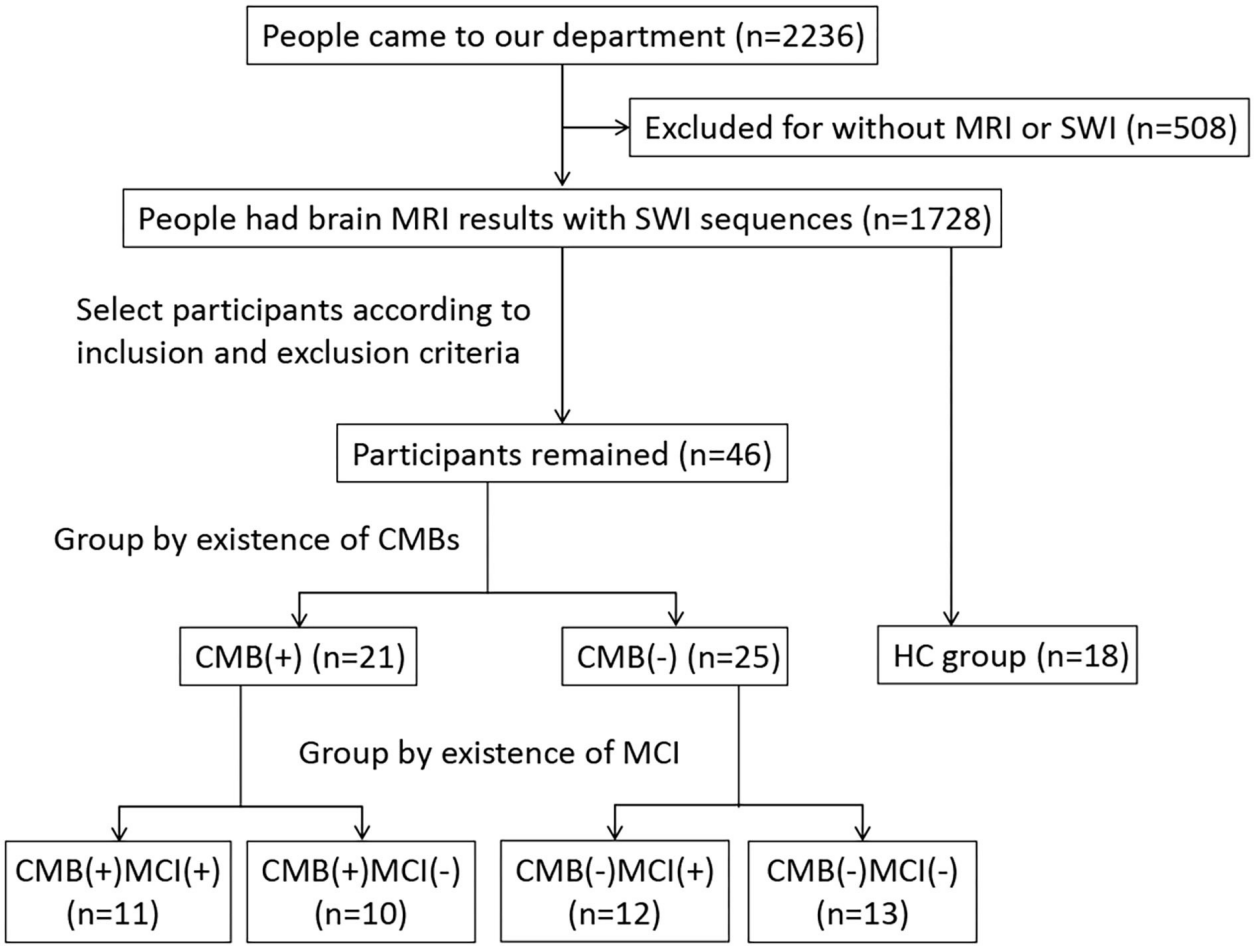
Flowchart of study population.

We conducted a cross-sectional study approved by the ethics committee and obtained informed consent from all participants. From August 2021 to March 2022, people who had imaging features of CSVD were included in the study and grouped by the presence of CMBs. We diagnosed CMBs and other CSVD features according to the criteria published in 2013 (Wardlaw et al., 2013). A CMB was defined as an ovoid or circle, homogeneous, well-circumscribed area of signal void in susceptibility-weighted imaging (SWI), with a diameter of no more than 10 mm. The signal intensity of perivascular space is similar to that of cerebrospinal fluid in all sequences, and the diameter of perivascular space is usually smaller than 3 mm. The irregular high signal in white matter on T2-weighted imaging (T2WI) and fluid-attenuated inversion recovery (FLAIR) was considered white matter hyperintensity (WMH). Recent small subcortical infarct is neuroimaging evidence of recent perforating arteriole infarction in diffusion-weighted imaging (DWI). Ultimately, we assigned 21 and 25 patients to the CMB(+) and CMB(–) groups, respectively. We also recruited 18 people with normal MRI results without neurological or other severe diseases as the healthy control (HC) group. All participants underwent MRI scanning (e.g., the sequence of T1WI, T2WI, FLAIR, DWI, and SWI) and got scores higher than 17 on the Montreal Cognitive Assessment (MoCA). All the participants were right-handed and older than 50 years. We excluded patients with diseases affecting cognition or iron accumulation, including cerebral infarction (except for a single acute infarction that did not occur in the thalamus, basal ganglia, and hippocampus), lesions occurring in rich-club nodes, cerebral hemorrhage, brain atrophy, brain tumor, brain injury, encephalitis, epilepsy, Parkinson's disease, dementia, multiple sclerosis, syphilis, AIDS, Creutzfeldt-Jakob disease, severe mental disorder, poisoning, hyperthyroid or hypothyroid, and history of ferralium use, alcoholism, or drug abuse. We also excluded patients who could not cooperate in cognitive assessment or MRI examination. Figure 1 shows the flowchart of the study participants.

### Cognitive assessment

Two professional neuropsychiatrists used the Beijing version of MoCA with a 1-point correction for people with <12 years of education (www.mocatest.org) to detect MCI. The maximum MoCA score is 30. A score ≥26 means normal cognitive function. We considered participants with scores of 18–25 as having MCI (Nasreddine et al., 2005). Based on the cognitive assessment results, we assigned 23 participants to the MCI(+) group (11 had CMBs) and the others to the MCI(–) group (10 had CMBs).

### MR imaging

We performed MRI scans with a Siemens Magnetom Aera 1.5 T MR scanner (Siemens Healthcare, Germany). We obtained the sequences of T1WI, T2WI, FLAIR, DWI, and SWI. The parameters of 3-D gradient-recalled-echo SWI sequence (e.g., magnitude image, phase image and SWI) were repetition time (TR) = 49 ms, echo time (TE) = 40 ms, flip angle = 15°, field of view (FOV) = 208 × 230 × 134 mm^3^, voxel size = 0.9 × 0.9 × 2 mm^3^, slice thickness = 2 mm, and slice number = 56. Other sequences used conventional parameters. We rated WMH severity using the Fazekas scale based on FLAIR. Total scores of periventricular and deep subscales range from 0 to 6, and each of them yielded a score of 0–3 (Fazekas et al., 1987).

### Image processing and analysis

**Figure 2:**
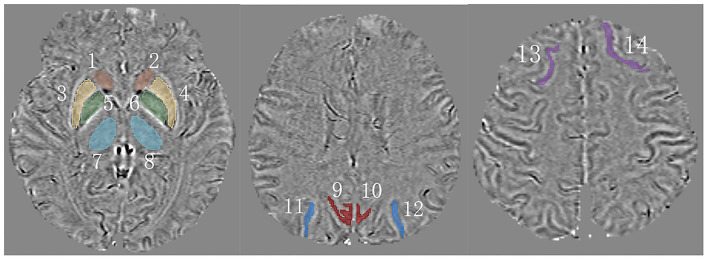
A schematic of measured regions on QSM. 1,2 = caudate nucleus (CN); 3,4 = putamen (PU); 5,6 = globus pallidus (GP); 7,8 = thalamus (TH); 9,10 = precuneus (PR); 11,12 = superior occipital gyrus (SOG); 13,14 = superior frontal gyrus (SFG).

To obtain QSM, we imported magnitude and phase images into STI Suite 3.0 (Medical Imaging, Brain Imaging, and Cell Modulation, University of California, Berkeley, CA, USA) based on MATLAB R2019a (Mathworks, Natick, MA, USA). First, we generated a phase mask in accordance with the thresholding of magnitude images. Second, we utilized a Laplacian-based method (HARPERELLA) for phase unwrapping and the 3-D variable-kernel Sophisticated Harmonic Artifact Reduction for Phase data (V-SHARP) method for background field removal (Özbay et al., 2017). Third, we performed Streaking Artifact Reduction (STAR) to yield QSM (Wei et al., 2015). Finally, we saved the QSMs as Digital Imaging and Communications in Medicine (DICOM) format files. Next, we opened our QSM data in 3-D Slicer (version 4.13.0; www.slicer.org) (Fedorov et al., 2012) and measured magnetic susceptibility values in regions of interest by manual contouring (Figure 2). Unfortunately, the coordinate system of Siemens is not a right-handed helix, which contraries MR scanning devices from other manufacturers like GE or Philips. Thus, our images restructured by the STI Suite software showed paramagnetic substances as lower signals, and diamagnetic substances were reversed. To tally with widespread means of expression, we multiplied our data by “−1.”

### Statistical analysis

We analyzed all data in our study using IBM SPSS Statistics for Windows (version 25.0; IBM Corp, Armonk, NY, USA) and produced all graphs using GraphPad Prism for Windows (version 9.0.0; San Diego, California, USA). We expressed continuous variables as mean ± standard deviation and median (interquartile range). We expressed categorical variables as frequency (%). We tested the data's normal distribution and homoscedasticity using Shapiro–Wilk's test (group sample < 50), Kolmogorov–Smirnov test (group sample > 50), and Levene's test. We compared the groups using a one-way analysis of variance (ANONA) for continuous variables and Scheffe's test for paired comparisons. We compared categorical variables through Pearson's chi-square test (expected frequencies > 5) or Fisher's exact test (expected frequencies <5) (the pairwise test used the *z*-test, Bonferroni correction: *p* < 0.05/7 = 0.007). We compared non-normally distributed data through the Mann–Whitney *U*-test (two groups) and Kruskal–Wallis test (k groups). After the above processing, we performed binary logistic regression to analyze the risk factors of MCI in patients with CMBs. The *p*-values <0.05 were considered statistically significant.

## Results

### The reliability of QSM

**Figure 3:**
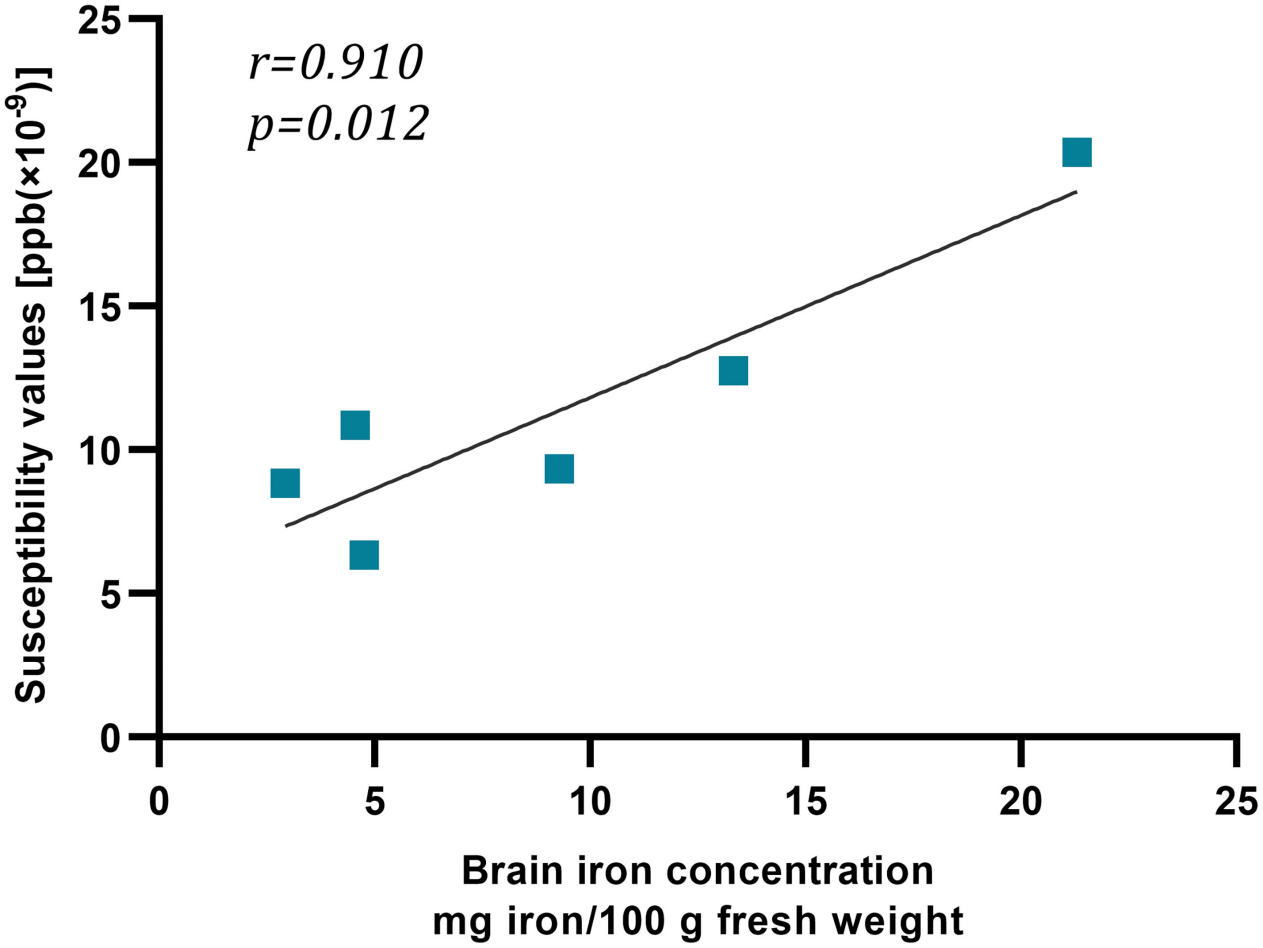
The relation between susceptibility values and iron concentration. This graph showed strong linear relation between susceptibility values [ppb (× 10^−9^)] and iron concentration (mg iron/100 g fresh weight).

We recruited 18 healthy people over the age of 50 years to investigate the relativity of susceptibility value and iron concentration. The standard of brain iron concentration referred to the postmortem research of Hallgren and Sourander (1958). The frontal cortex, caudate nucleus, putamen, globus pallidus, thalamus, and occipital cortex contained, on average, 2.92, 9.28, 13.32, 21.30, 4.76, and 4.55 mg iron/100 g fresh weight, respectively. Susceptibility values of above 6 regions were 8.85, 9.34, 12.76, 20.36, 6.35, and 10.86 (× 10^−9^ ppb). Iron concentration and susceptibility values appeared to be correlated, indicating the reliability of QSM and our measuring method (*r* = 0.910, *p* = 0.012) (Figure 3).

### Clinical characteristic

We first compare the characteristics of CMB(+), CMB(–), and HC groups. Only MoCA scores were statistically different (*p* < 0.001). Scheffe's test revealed significant differences between HC and the other two groups (vs. CMB(+): *p* < 0.001; vs. CMB(–): *p* = 0.007) (Table 1). When we subdivided the CMB(+) and CMB(–) groups by cognitive impairment, Fazekas scores showed differences (*p* = 0.045) but no statistical significance after paired comparison [CMB(+)MCI(–) vs. CMB(–)MCI(–): *p* = 0.118; CMB(–)MCI(+) vs. CMB(–)MCI(–): *p* = 0.094; CMB(+)MCI(+) vs. CMB(–)MCI(–): *p* = 0.303; the rest of the pairwise tests: *p* = 1.000] (Table 2).

**Table 1:** The characteristics of participants with or without CMB.

**Variable**	**CMB(+) (*n* = 21)**	**CMB(–) (*n* = 25)**	**HC (*n* = 18)**	***p*-value**
Age (years)^1^	63.86 ± 7.93	65.72 ± 7.47	63.33 ± 10.03	0.611
Male^2^	9 (42.9%)	10 (40.0%)	10 (55.6%)	0.578
Hypertension^2^	14 (66.7%)	13 (52.0%)	–	0.377
Diabetes mellitus^2^	5 (23.8%)	5 (20.0%)	–	1.000
Hyperlipidemia^2^	11 (52.4%)	9 (36.0%)	–	0.372
Hemoglobin (g/L)^1^	130.43 ± 10.39	134.44 ± 15.31	135.28 ± 16.22	0.507
Smoking^2^	4 (19.0%)	7 (28.0%)	1 (5.6%)	0.189
Education (years)^3^	9 (8, 12)	9 (9, 11)	9 (7, 11)	0.845
BMI (kg/m^3^)^3^	24.98 (23.37, 26.43)	24.86 (23.80, 25.98)	24.75 (22.78, 26.29)	0.942
RSSI^2^	2 (9.5%)	1 (4%)	–	0.585
WMH	21 (100.0%)	25 (100.0%)	–	NA
Fazekas Scores^4^	2 (1, 2)	2 (1.5, 3)	–	0.153
PVS^2^	14 (66.7%)	17 (68.0%)	–	1.000
MoCA^1^	24.52 ± 3.06	25.44 ± 2.76	28.06 ± 1.55	<0.001^b^^,^^c^

PVS, Perivascular space; RSSI, Recent small subcortical infarct; WMH, White matter hyperintensity.

Normal distribution data were expressed as mean ± standard deviation; abnormal distribution data were expressed as median (interquartile range); categorical variables were expressed as frequency (%).

Statistical method:

1 one-way analysis of variance test (pairwise test used Scheffe's test).

2 Pearson's chi-square test (expected frequencies > 5) or Fisher's exact test (expected frequencies <5), pairwise test used the z-test, Bonferroni correction: p < 0.05/7 = 0.007.

3 Kruskal–Wallis test (multiple comparison used all pairwise).

4 Mann–Whitney U-test.

Pair–wise test:

^a^p-value of CMB(+) vs. CMB(–) < 0.05,

b p-value of CMB(+) vs. HC < 0.05,

c p-value of CMB(–) vs. HC << 0.05.

**Table 2:** The characteristics of participants with or without MCI.

**Variable**	**CMB(**+**) (*****n*** = **21)**		**CMB(–) (*****n*** = **25)**		***p*-value**
	**MCI(+) (*n* = 11)**	**MCI(–) (*n* = 10)**	**MCI(+) (*n* = 12)**	**MCI(–) (*n* = 13)**	
Age (years)^1^	64.45 ± 6.11	63.20 ± 9.87	65.67 ± 6.83	65.77 ± 8.30	0.854
Male^2^	7 (63.6%)	5 (50%)	8 (66.7%)	7 (53.8%)	0.857
Hypertension^2^	9 (81.8%)	5 (50%)	5 (41.7%)	8 (61.5%)	0.256
Diabetes mellitus^2^	3 (27.3%)	2 (20%)	2 (16.7%)	3 (23.1%)	0.962
Hyperlipidemia^2^	6 (54.5%)	5 (50%)	4 (33.3%)	5 (38.5%)	0.740
Hemoglobin (g/L)^1^	135.09 ± 7.71	125.30 ± 10.86	131.33 ± 18.86	137.31 ± 11.14	0.162
Smoking^2^	1 (9.1%)	3 (30%)	4 (33.3%)	3 (23.1%0	0.550
Education (years)^1^	8.09 ± 3.78	10.30 ± 2.45	8.46 ± 1.95	10.23 ± 1.88	0.093
BMI (kg/m^2^)^3^	25.00 (23.53, 26.64)	24.49 (22.79, 26.50)	24.93 (23.55, 26.40)	24.22 (23.87, 25.77)	0.969
RSSI^2^	1 (9.1%)	1 (10%)	1 (8.3%)	0 (0%)	0.690
WMH	11 (100%)	10 (100%)	12 (100%)	13 (100%)	NA
Fazekas Scores^3^	2 (1, 2)	2 (1, 2)	2 (1, 2)	3 (2, 4)	0.045
PVS^2^	7 (63.6%)	7 (70%)	9 (75%)	8 (61.5%)	0.924
The number of CMB^4^	1 (1, 2)	1 (1, 1)	–	–	0.314

Normal distribution data were expressed as mean ± standard deviation; abnormal distribution data were expressed as median (interquartile range); categorical variables were expressed as frequency (%).

Statistical method:

1 one-way analysis of variance test (pair–wise test used Scheffe' s test),

2 Fisher's exact test (pairwise test used the z-test, Bonferroni correction: p < 0.05/7 = 0.007),

3 Kruskal–Wallis test (multiple comparison used all pairwise).

4 Mann– Whitney U-test.

Pairwise test results: Fazekas scores were different (p = 0.045) but paired comparison revealed that these were not significant (CMB(+)MCI(–) vs. CMB(–)MCI(–), p = 0.118; CMB(–)MCI(+) vs. CMB(–)MCI(–), p = 0.094; CMB(+)MCI(+) vs. CMB(–)MCI(–), p = 0.303; the rest of pairwise tests p = 1.000).

### Iron deposition in rich-club nodes

**Figure 4:**
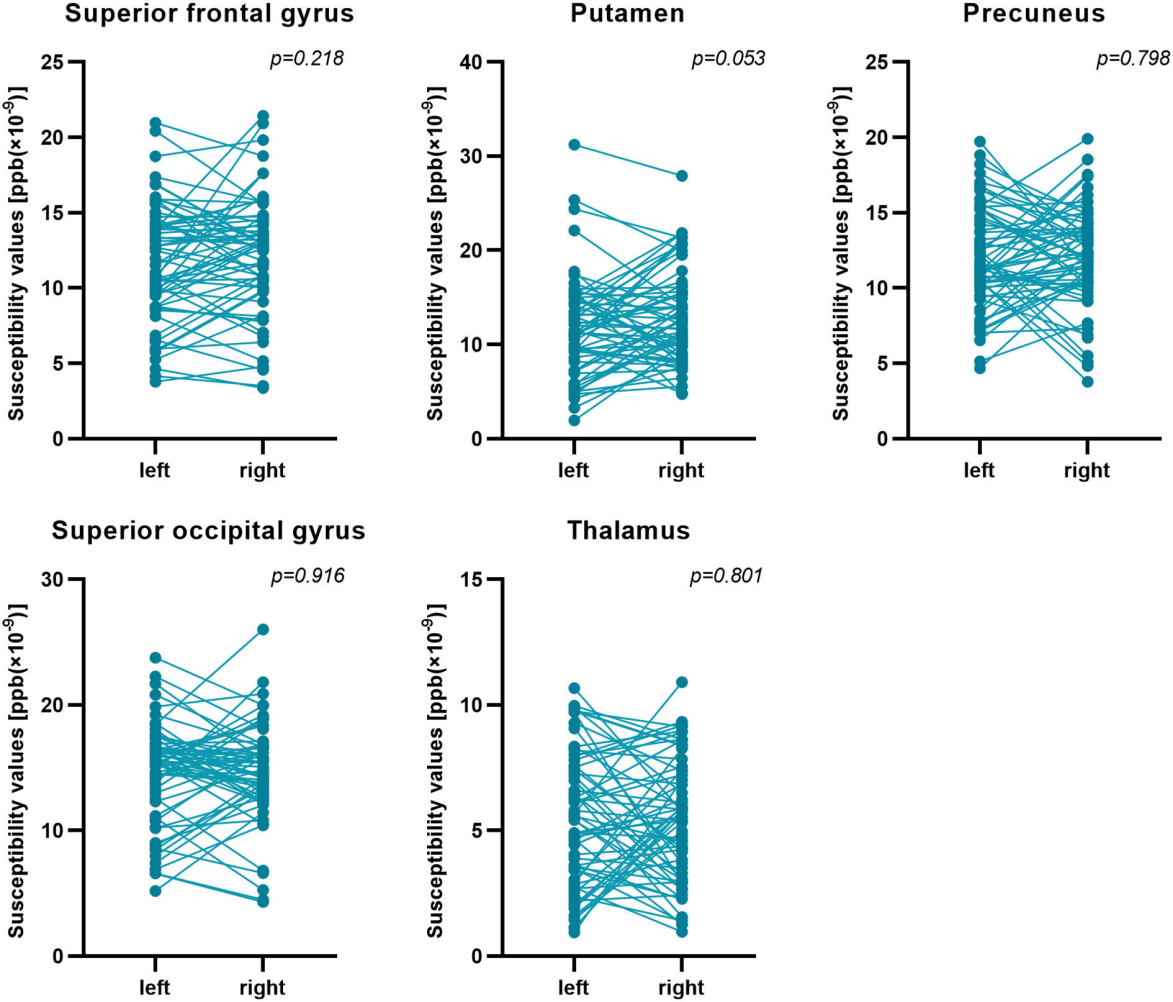
Susceptibility values of bilateral hemispheres. Comparison of the susceptibility values of all participants' bilateral hemispheres by paired *t-*test. There was no significant difference between them.

**Figure 5:**
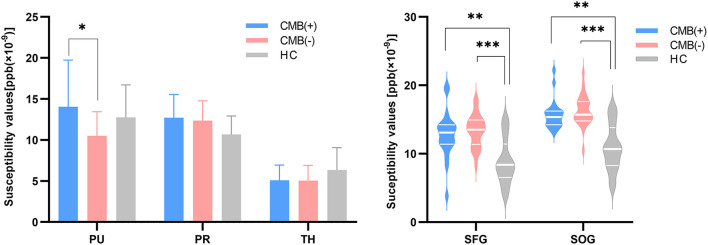
The iron content of rich-club nodes (grouped by the presence of CMB). The histogram expresses normal distribution data; “T” denotes standard deviation. The violin diagram expresses abnormal distribution data; three transverse lines denote the 75th percentile, median, and 25th percentile (top to bottom). *p-value < 0.05, **p-value < 0.01, ***p-value < 0.001.

**Figure 6:**
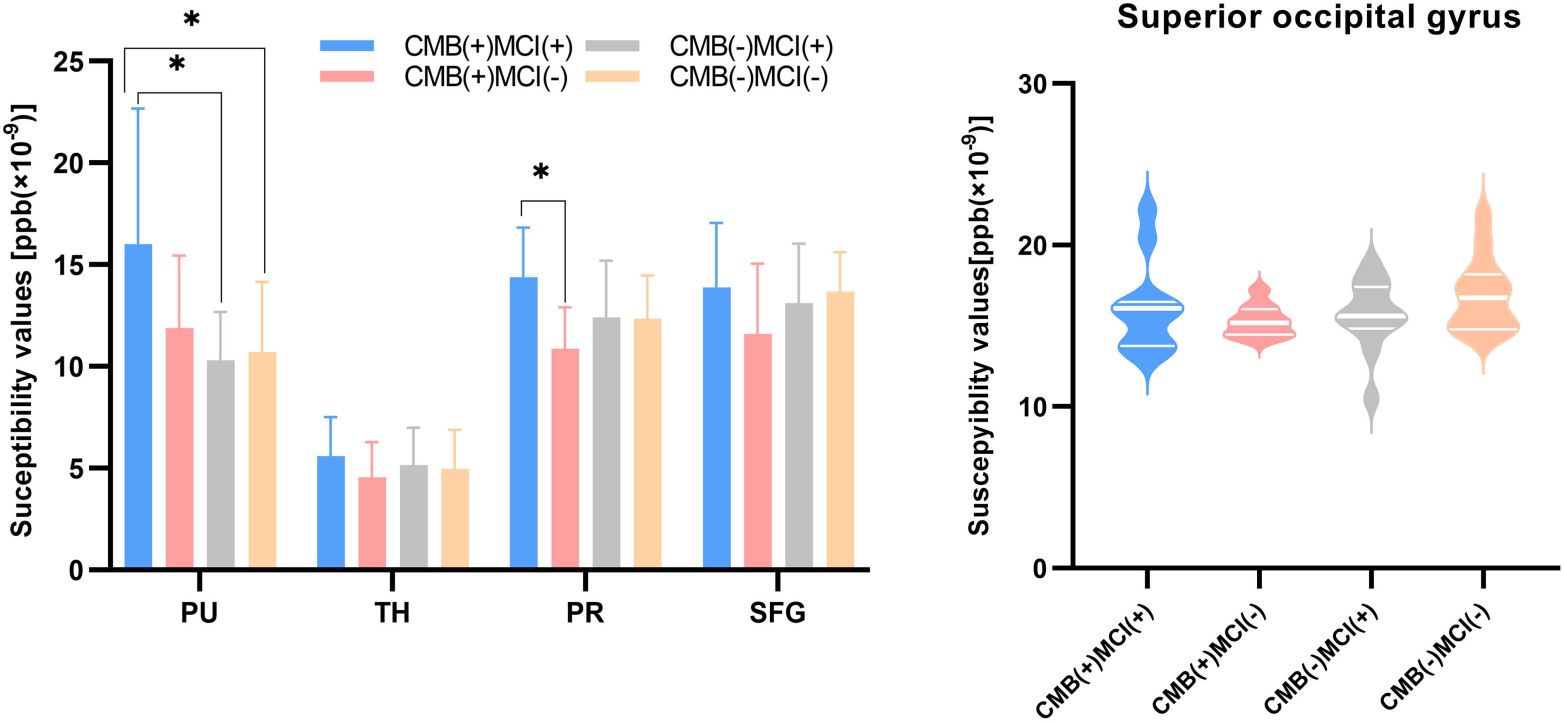
The iron content of rich-club nodes (grouped by the presence of CMB or MCI). The histogram expresses normal distribution data; “T” denotes standard deviation. The violin diagram expresses abnormal distribution data; three transverse lines denote the 75th percentile, median, and 25th percentile (top to bottom). *p-value < 0.05.

First, the 64 participants had similar susceptibility values in bilateral hemispheres (Figure 4). Thus, we analyzed rear data based on the means of bilateral values. There were 21 participants in the CMB(+) group, 25 in the CMB(–) group, and 18 in the HC group. Among the rich-club nodes, the putamen of the CMB(+) and CMB(–) groups had significantly different susceptibility values (*p* = 0.029). The susceptibility values of SFG and SOG of the CMB(–) and HC groups were significantly different (SFG: *p* < 0.001; SOG: *P* < 0.001), as well as those of the CMB(+) and HC groups (SFG: *p* = 0.008; SOG: *p* = 0.002) (Figure 5). After the cognitive assessment, we subdivided the the CMB (+) and CMB (−) groups and found significant differences in the precuneus susceptibility values of the CMB(+)MCI(+) and CMB(+)MCI(–) groups (*p* = 0.016). The susceptibility values of the putamen were also significantly different in the CMB(+)MCI(+) group and the two CMB(+) groups (MCI(+): *p* = 0.026; MCI(–): *p* = 0.038) (Figure 6). Next, binary logistic regression analysis (backward: LR) of the susceptibility values of the putamen and precuneus revealed that only higher susceptibility values of precuneus affected cognitive function in patients with CMBs (odds ratio = 1.937, 95% confidence interval: 1.146–3.276, *p* = 0.014) (Table 3).

**Table 3:** The binary logistic regression analysis (backward: LR) results in iron deposition and cognitive impairment.

**(A). CMB(**+**) group**									
	**Factors**	**B**	**SE**	**Wald χ^2^**	**Freedom**	***p*-value**	**Exp (B)**	**95%CI**	
								**Minimum**	**Maximum**
Step 1		−10.619	4.455	5.681	1	0.017	0.000		
	Putamen	0.198	0.165	1.444	1	0.229	1.219	0.883	1.683
	Precuneus	0.647	0.280	5.323	1	0.021	1.909	1.102	3.307
Step 2		−8.241	3.400	5.874	1	0.015	0.000		
	Precuneus	0.661	0.268	6.090	1	0.014	1.937	1.146	3.276
**(B). CMB(–) group**									
	**Factors**	**B**	**SE**	**Wald** χ^2^	**Freedom**	***p*** **-value**	**Exp (B)**	**95%CI**	
								**Minimum**	**Maximum**
Step 1		0.268	2.561	0.011	1	0.917	1.307		
	Putamen	−0.047	0.140	0.111	1	0.739	0.954	0.725	1.256
	Precuneus	0.012	0.169	0.005	1	0.946	1.012	0.726	1.410
Step 2		0.408	1.524	0.072	1	0.789	1.504		
	Putamen	−0.046	0.140	0.110	1	0.740	0.955	0.725	1.256
Step 3		−0.080	0.400	0.040	1	0.842	0.923		

## Discussion

Our results suggest that patients with CMBs accumulate more iron in the putamen than individuals without CMBs and that iron deposition in SFG and SOG in CSVD patients without CMBs differs from that in healthy individuals. In the CMB group, iron deposition in the precuneus emerged as an independent risk factor of MCI. Besides, we confirmed that CMBs might influence iron content in remote rich-club nodes and be associated with cognitive impairment.

Regarding iron accumulation in deep gray matter, we found statistically significant differences between the CMB(+) and CMB(–) groups and no significant difference between the CMB(–) and HC groups. These results are consistent with other studies (Valdés Hernández et al., 2016; Li et al., 2022). Besides, we observed higher iron content in the thalamus of HCs than in CMB(–) patients who all had WMH although it is not significant. This observation is in line with the study of Gattringer et al. (2016), indicating that WMH did not induce iron deposition in deep gray matter and even reduced it in patients with severe WMH. However, the specific mechanism remains unclear. In addition, both the CMB(+) and CMB(–) groups showed higher susceptibility values in SFG and SOG than in the HC group. Meanwhile, latent iron-mediated damage in the cortex among patients with CSVD might exist, and investigators should, thus, consider the cortex but not merely deep gray matter.

A prior study considered that the CMB count was associated with cognitive impairment (Akoudad et al., 2016). The CMB(+)MCI(+) and CMB(+)MCI(–) groups had similar CMB counts in our study. This result was probably due to slight cognitive impairment, and the low total burden of CSVD leads to CMB counts ranging from one to three. Both Akoudad et al. (2013) and Liu et al. (2020) considered that a single CMB could reduce white matter integrity. Our study demonstrated that less-severe CMB could still cause iron deposition in distant regions, which may lead to cognitive impairment.

Alberto et al. discovered that removing the interference of vascular risk factors (e.g., hypertension, hyperlipidemia, diabetes, and smoking) suppressed the association between Fazekas scores and MCI. Nevertheless, Alzheimer's disease was still related to WMH severity (Mimenza Alvarado et al., 2018). All of our patients had slight cognitive impairment and similar vascular risk factors. This might explain why all groups had similar Fazekas scores.

Previous studies have mainly focused on lesions occurring in rich-club nodes. We showed that even if lesions were distant to rich-club nodes of CSVD, iron deposition occurs in the precuneus (one of the rich-club nodes) and might cause cognitive impairment. Thus, CSVD damage is not only in the focal but also widespread in the whole brain (Ter Telgte et al., 2018). Furthermore, the precuneus is a major hub of the default-mode network playing a crucial role in attention and executive function (Dey et al., 2016). Previous study has shown that CMBs were associated with impairments in processing speed, visuospatial performances, and executive function (Li et al., 2021; Cipriano et al., 2022). Our results perhaps could be interpreted by these theories.

Patients in our study did not suffer from brain atrophy. Yet we observed iron deposition in their cortex, which is in line with the theory stating that iron deposition appears before cortex atrophy (Rodrigue et al., 2013; Sánchez-Castañeda et al., 2015), although confirming this requires longitudinal research.

Cerebral microbleed is a kind of imaging diagnosis. CMBs have many etiologies. Cerebral amyloid angiopathy (CAA) is the most common reason for cerebral lobar microbleeds (Renard, 2018). Thus, the CMB(+) group might contain some patients with CAA. The pathological feature of CAA is amyloid β (Aβ) peptide accumulation in the walls of arterioles and leptomeninges, which might induce microbleeds by degenerating the smooth muscle cells of vessels and remodeling vessels walls (Charidimou et al., 2017). Previous studies have confirmed the co-localization of Aβ and iron deposition (van Bergen et al., 2016; Ayton et al., 2017). Moreover, Aβ accumulation in the precuneus might impair the default-mode network (Mormino et al., 2011). However, we did not perform positron emission tomography/computed tomography to confirm whether the location of iron deposition overlapped with that of Aβ. If they are co-localized, our results suggest that Aβ-induced cerebral lobar microbleeds might coexist with the buildup of Aβ in the cortex, thereby assuming iron deposition in the cortex, away from CMBs. If they are not co-localized, the following paragraph describes a possible mechanism.

As far as is currently known, CMBs are similar to lacunar infarcts and WMH in that they can affect the cortex by decreasing the amount and intensity of connections in the structure network (Liu et al., 2020). According to the remote effect of cerebral infarction, we want to propose a hypothesis (Linck et al., 2019). Local microhemorrhage induces red cell lysis and releases hemoglobin. As a degradation product, iron impairs neurons *via* oxidative stress (Yang et al., 2021). It reduces white matter integrity (Liu et al., 2020), which may disorder signal transmission among neurons and stimulate rich-club nodes, thereby damaging them. Dying neurons, surrounding macrophages and microglia, release iron that accumulates and aggravates hazards in rich-club nodes (Vernooij, 2019). Finally, the cognitive function begins to decline. However, confirming this mechanism requires empirical evidence.

Besides, our study has some limitations. First, this is a cross-sectional study, so we cannot prove the causality between CMBs, iron deposition, and cognitive impairment. Second, this small sample single-center study could not include patients of all ages, etiologies, and regions. Third, we did not acquire comprehensive assessments for specific cognitive domains. Provided that prospective researchers utilize susceptibility tensor imaging (STI) combined with the location of CMB to investigate particular pathways and effects of iron, it will supply further evidence. Besides, a larger sample size, grouping by etiologies, and long-term follow-up are warranted.

## Data availability statement

The raw data supporting the conclusions of this article will be made available by the authors, without undue reservation.

## Ethics statement

The studies involving human participants were reviewed and approved by the Human Research Ethics Committee of the First Affiliated Hospital of Xi'an Medical University. The patients/participants provided their written informed consent to participate in this study.

## Author contributions

JT: formulation, collection, processing, analysis, and writing the original draft. JY: imaging technology support. DL and XW: neuropsychological assessment. JL: research formulation. FG: supervision, reviewing, and editing the manuscript. All authors contributed to the article and approved the submitted version.

## Funding

This work was supported by a grant from The First Affiliated Hospital of Xi'an Medical University (No. XYFYPT-2020-03).

## Conflict of interest

The authors declare that the research was conducted in the absence of any commercial or financial relationships that could be construed as a potential conflict of interest.

## Publisher's note

All claims expressed in this article are solely those of the authors and do not necessarily represent those of their affiliated organizations, or those of the publisher, the editors and the reviewers. Any product that may be evaluated in this article, or claim that may be made by its manufacturer, is not guaranteed or endorsed by the publisher.

## Abbreviations

CSVD: cerebral small vessel disease
CMBs: cerebral microbleeds
MCI: mild cognitive impairment
MRI: magnetic resonance imaging
SFG: superior frontal gyrus
SOG: superior occipital gyrus
CN: caudate nucleus
PU: putamen
GP: globus pallidus
TH: thalamus
PR: precuneus
T1WI: T1-weighted imaging
T2WI: T2-weighted imaging
DWI: diffusion weighted imaging
FLAIR: fluid-attenuated inversion recovery
SWI: susceptibility weighted imaging.
